# Perception of strong social norms during the COVID-19 pandemic is linked to positive psychological outcomes

**DOI:** 10.1186/s12889-022-13744-2

**Published:** 2022-07-22

**Authors:** Shuang Liu, Jiajia Zhu, Yutong Liu, Danica Wilbanks, Joshua Conrad Jackson, Yan Mu

**Affiliations:** 1 CAS Key Laboratory of Behavioral Science, Institute of Psychology, Chinese Academy of Sciences, 16 Lincui Road, Chaoyang District, Beijing, 100101 China; 2 Department of Psychology, University of Chinese Academy of Sciences, Beijing, 100049 China; 3 Department of Psychology and Neuroscience, University of North Carolina at Chapel Hill, Chapel Hill, NC 27599 USA; 4 Kellogg School of Management, Northwestern University, Evanston, IL 60208 USA

**Keywords:** COVID-19, Cultural tightness-looseness, Social norms, Public mental health, Trust

## Abstract

Social norms can coordinate individuals and groups during collective threats. Pandemic-related social norms (e.g., wearing masks, social distancing) emerged to curb the spread of COVID-19. However, little is known about the psychological consequences of the emerging norms. We conducted three experiments cross-culturally, during the early period of the COVID-19 pandemic in China (Study 1), the recovery period in China (Study 2), and the severe period in the United States and Canada (Study 3). Across the three studies, we first distinguished the opposite effects of social norms and risk perception on individuals’ psychological characteristics during the COVID-19 pandemic and further revealed that individuals who perceived stronger pandemic norms reported a lower level of COVID-19 risk perception, which in turn would be associated with fewer negative emotions, lower pressure, more positive emotions, higher levels of trusts, and more confidence in fighting against COVID-19. Our findings show that perceived tighter social norms are linked to beneficial psychological outcomes. This research helps governments, institutions, and individuals understand the mechanism and benefits of social norms during the pandemic, thereby facilitating policy formulation and better responses to social crises.

**Supplementary Information:**

The online version contains supplementary material available at 10.1186/s12889-022-13744-2.

## Introduction

COVID-19 has dramatically impacted people worldwide, with over 500 million reported cases and more than 6,277,241 deaths as of May 17, 2022 (https://coronavirus.jhu.edu/). Moreover, the COVID-19 pandemic puts immense psychological burdens on individuals and society [1]. For example, an online survey with 1210 Chinese people during the initial stage of the COVID-19 pandemic showed that 54% of respondents rated the psychological impact of the COVID-19 pandemic as moderate or severe and that 29% reported moderate to severe anxiety symptoms [2]. Evidence from 5065 American adults suggests that as the pandemic continued, each additional day was significantly associated with an 11% increase in the odds of moving up a category of distress among the high-count states (i.e., New York with 50 or more COVID-19 cases as of March 10, 2020) [3]. People’s psychological well-being was in a vulnerable state during the pandemic, with higher risk sensitivity, more negative emotions, and more frequent interpersonal problems [3–7]. Worldwide research demonstrated that people who felt greater pandemic risk had poorer mental health during the pandemic, with a higher rate of depression, more perceived stress and anxiety, and a higher frequency of preventive behaviors such as washing hands [8–13]. However, little attention has been paid to whether changing contextual factors (i.e., social norms) may have influenced people’s psychological wellbeing and social attitudes during the global pandemic.

Tightness-Looseness (TL) theory defines social norms as socially agreed-upon standards of behaviors which vary in their strength and enforcement across cultures [14]. Tighter societies with stronger social norms (e.g., China, Japan, South Korea, Germany), have higher levels of social order, government control, solidarity, and coordination; societies with looser cultures (e.g., the United States, Canada, Greece, ﻿Netherlands), have more freedom and openness [15–17]. Tight cultures tend to have higher historical rates of ecological threats, and some evidence suggests that tightness can help groups coordinate to survive under high threat [17, 18]. Previous evolutionary game theory (EGT) modeling work has simulated the evolutionary process of norm adherence and enforcement behavior under different levels of threat and found that stronger norms are more evolutionarily adaptive when facing high collective threat [1, 18].

To fight against COVID-19, most nations have promoted new social norms (e.g., wearing masks when going out, washing hands frequently, and keeping social distance). These emerging social norms have proven to help decrease viral transmission speed during the COVID-19 pandemic and reduce risk of infection [19–22]. For instance, Gelfand et al. conducted a global analysis of the effect of culture TL (measured *before the pandemic*) on COVID-19 cases and deaths [23]. They found that nations with low levels of TL were estimated to have 4.99 times the number of cases and 8.71 times the number of deaths — even after controlling for important covariates like the national economy, population, median age, and government efficiency. Therefore, we propose that social norms during the pandemic have a positive influence on reducing COVID-19 risk thereby improving psychological outcomes.

In sum, previous research has shown that (a) the COVID-19 pandemic has had a range of negative mental health implications, and (b) strong social norms before the pandemic have been instrumental for slowing the spread of COVID-19 cases. However, there is no direct evidence showing whether and how the strength of social norms *during the pandemic* affects people’s psychological well-being and attitudes. The current study intends to bridge the gap between these two streams of evidence by investigating whether individual-level perceived strength of social norms can ameliorate some of the adverse psychological consequences of the pandemic.

Considering COVID-19 has become a sustained global crisis, pandemic social norms will likely persist for some time. Therefore, the current study set out to uncover the direct and indirect effects of adherence to these social norms on psychological outcomes during the COVID-19 pandemic. We conducted three studies in different cultural groups during the early period of the COVID-19 pandemic in China (Study 1), the recovery period of COVID-19 in China (Study 2), and the severe period of COVID-19 in the United States and Canada (Study 3).

The goal of Study 1 was to test the association between the perceived strength of social norms during the pandemic and psychological outcomes, and the mediating role of risk perception. We collected data using a large-scale survey from January 30th to February 3rd, 2020 — a period when the COVID-19 pandemic in China was severe and the WHO declared a global alert for COVID-19 [24]. All Chinese provinces entered the highest level of public health response (Level 1 of 4 levels in the Chinese Emergency System) [25]. During this time, new social norms related to pandemic prevention emerged in the public like staying at home, washing hands, wearing masks when going out, and alcohol sterilization. We first tested the hypothesis about the adverse effect of COVID-19 risk on both people’s psychological well-being and social attitudes. Then, we set out to examine the link between pandemic norms and psychological consequences (i.e., psychological well-being and social attitudes). As aforementioned, historically tight cultures have experienced higher levels of ecological and social threats, and people in tight cultures have greater obedience to social norms. Therefore, we hypothesize that (1a) the perceived strength of social norms during the pandemic would be positively related to people’s psychological well-being, that is, perceived strength of social norms would be associated with more positive emotions, fewer negative emotions, and lower pressure; (1b) the perceived strength of social norms during the pandemic would predict people’s social attitudes, that is, people who perceived stronger social norms would also show higher trust in organizations and other people. Additionally, considering the function of social norms in facilitating coordination under threats, we hypothesized that the perceived strength of social norms would be associated with lower perception of risk which in turn would be associated with better mental health and more trust in institutions.

To further validate our findings, in Study 2, we collected two waves of data during the recovery period of the COVID-19 pandemic in China, from May 2nd to May 7th, 2020, and from July 6th to July 15th, 2020. We expected to replicate Study 1's findings in this follow-up survey.

Since China is a tighter culture (with a standardized tightness score of .19 according to [26]), we were also curious about whether findings from data collected in China would still hold in loose cultures. In Study 3, therefore, we set out to replicate our findings in two culturally loose countries — the United States of America and Canada, which have standardized tightness scores of −.13 (US) and − .14 (Canada) [26].

## Methods

### Participants

In Study 1, we distributed a set of questionnaires through an online platform similar to Qualtrics from January 30th to February 3rd, 2020. For large-scale sampling, we pre-calculated the sample size by the widely accepted Monte Carlo method invented by Schoemann and her colleagues [27] (see Supplementary Methods for Sample Size and Power Analysis). We set an automatic filter to remove invalid data and careless responses through multiple probe items according to recommended reprocessing methods [28]. Thus, we obtained valid data from 1179 participants who correctly answered the probe questions. Data from 18 participants not living in mainland China within the past 6 months were excluded. We set mandatory options on all key variables, so we did not further exclude data based on missing values. The final sample was 1161 (350 males, 661 females, and 150 not providing; mean age 34.3 ± 10.62), covering 31 provinces, Municipalities, and Autonomous Regions of mainland China (except Hong Kong, Macao, and Taiwan). All participants gave their informed consent and volunteered to participate in the survey without compensation.

In Study 2, according to Monte Carlo methods for mediation analyses [27], we obtained the minimum required sample size of 100 to reach a power level of .90 (Supplementary Methods). We obtained 348 total respondents (1st round: 233; 2nd round: 115). We excluded data with wrong answers in the probe question (*N* = 28) and invalid data (only filled out the informed consent part of the questionnaire) (*N* = 5). We also excluded data with a short completion time (1.5*SD* below the mean, 2493.94–1.5*1337.76 seconds) (*N* = 8). Since we didn’t find significant differences in demographic information, TL, perceived norms during the pandemic, and related mentalities between the two waves of the data (Table S1), we collapsed the two waves of data. The final sample in Study 2 was 307 (117 males and 190 females, mean age 24.65 ± 7.13 years) from 11 Chinese provinces. All participants gave their informed consent before the survey and received compensation for participation.

In Study 3, we recruited participants from the United States and Canada through an online participant recruitment platform (https://www.prolific.co/) respectively on May 3rd and May 20th, 2020 — a severe period of COVID-19. We followed the same minimum required sample size requirement calculated in Study 2 to recruit enough participants (Supplementary Methods). A total of 156 American participants completed the survey, 7 of which failed to pass the probe question. 149 Canadian participants answered the questionnaire, 10 of which failed to pass the probe question. Taken together, the final sample was 149 (89 males and 60 females, mean age 35.50 ± 12.99 years) and 139 (77 males and 62 females, mean age 30.90 ± 10.20 years) for the American and Canadian samples respectively.

For the three studies, we adopted the strategy that if the omitted missing values were related to demographic information (i.e., age, gender), we kept and reported them in the results. But if they were from the core variables, we excluded them in the following analyses instead of interpolating them. The present research was approved by the ethics committees of the Institute of Psychology, Chinese Academy of Sciences.

### Measures

Our measures were kept consistent across the three sub-studies. These included the perceived strength of social norms, risk perception, and two aspects of psychological outcomes (psychological well-being and social attitudes). For most of the variables, we used multiple established measures to increase the validity of our research. We included emotions, psychological pressure, and self-confidence respectively as indicators of psychological well-being, and measured organizational trust, interpersonal trust, and confidence in groups and organizations during the pandemic as indicators for social attitudes. We described more details about our measures below.

### Study 1

#### Perceived social norms

We measured the participants’ perceptions of social norms before and during the pandemic on a 7-point scale (1 = Strongly disagree, 7 = Strongly agree). For perceived strength of social norms in each of the two separate conditions, we used two items that were adapted from the Generalized Cultural Tightness-Looseness (TL) Scale [15], i.e., “during the pandemic/generally speaking, my habitual residence has a set of clear norms to restrict and guide people’s behavior and performance”, “during the pandemic/generally speaking, if people violate the regulations related to the pandemic in my habitual residence, others will be strongly against”. A higher score on the scale indicates that people perceived stricter social norms during the pandemic, or in general, respectively. The Cronbach’s α coefficients were .63 (during the pandemic) and .68 (in general).

#### Pandemic risk perception

We used one item for assessing people’s risk perceptions — the possibility of being infected by COVID-19 on a 6-point scale (1 = Very likely, 6 = Very unlikely), e.g. “How likely do you think you are infected with COVID-19?”. We reversed the score. The higher score indicates more perceived risk during the COVID-19 pandemic.

### Psychological well-being and social attitudes

#### Emotions

Adapting from the 60-item version of The Positive and Negative Affect Schedule (PANAS) [29], we asked participants to report their current emotions, including two positive emotions (i.e., happy, hopeful) and seven negative emotions (i.e., upset, anxious, scared, worried, tired, confused and angry). They were scored on a 4-point scale from 0 = None to 3 = Very strong. In later analyses, we calculated the mean score of the positive emotions and the mean of the negative emotions as the indicators for emotions. The Cronbach’s α coefficients were .48 and .86 respectively.

#### Pressure

Participants were asked to rate their pressure (“What is your psychological pressure currently”) from 0 (None) to 100 (very much). Higher scores correspond to higher pressure felt during the pandemic.

#### Organizational trust

We measured people’s trust in organizations using a scale which we adapted from the *Organizational Trust Scale* [30]. The scale included two dimensions: trust in authoritative institutions or groups and trust in non-authoritative institutions or groups. We included five items for trust in authorities, such as trust in the central and local government, trust in neighborhood offices, grassroots organizations, and neighborhood committees, trust in the World Health Organization, and trust in TV broadcasts and newspapers; and four items for trust in non-authorities, including trust in internet celebrities, trust in online social networks (e.g., WeChat moments and Tencent QQ groups), trust in relatives, friends, and neighbors, and trust in foreign media. Participants were required to rate their trust for those institutions and groups during the pandemic from 1 (Completely not trust) to 7 (Completely trust). We used the mean score of the two subscales (authoritative trust and non-authoritative trust) and the mean score of all items (organizational trust) together. Higher mean scores indicate a higher level of trust. The Cronbach’s α of the subscale of authoritative trust was .81; the Cronbach’s α of the subscale of non-authoritative trust was .66.

#### Demographic variables

Besides general demographic information like gender, age, education level, and profession, we collected participants’ geographical information, including their permanent residence, and temporary location when completing questionnaires.

### Study 2

To be consistent with Study 1, we included four kinds of measures: (1) individual-level perceived tightness of social norms: social norms during and before the pandemic; (2) pandemic risk perception; (3) pandemic related attitudes and feelings: personal feelings and attitudes toward themselves (incl., positive and negative emotions, pressure) and other people or organizations (incl., COVID-19 stigma, organizational trust, and interpersonal trust). We included additional measures of confidence in COVID-19 pandemic control of themselves, other people, and institutions; (4) participant demographic information.

#### Perceived social norms

Similarly, as in Study 1, we measured the perception of social norms before and during the pandemic on 7-point scales (1 = strongly disagree, 7 = strongly agree). For the perceived social norms during the pandemic, the scale was modified from [15] that of Study 1 and was extended into four items. The Cronbach’s α coefficient was .61. For the perceived social norms before the pandemic, we used the 15-item Daily TL Scale. It was adapted from the Latitude vs. Constraint in Daily Life Scale [31], assessing the frequency with which individuals are chronically exposed to a wide range of deviant behaviors, and a lack of conformity in their daily lives, e.g., “Is there a lot of littering in the street?”, “Do people wear informal clothing in public?”. Participants were asked to rate the frequency of each statement based on their daily experience from 1 (not at all) to 7 (very frequently). It was equivalent to the Generalized TL Scale but focused on the tightness-looseness of everyday life in public settings. The correlation between Daily TL and Generalized TL was significantly positive (*r* (305) = .52, *p* < .001). The higher the score, the more often people perceived constraints in their daily life. The Cronbach’s α coefficient for the scale was .81.

#### Pandemic risk perception

To measure risk perception, apart from one single item same as that of Study 1 (assessing the possibility of being infected by COVID-19 by 5-point scale), we developed a Risk Perception Scale in this study. We reasoned  that the degree of risk that COVID-19 caused was closely related to the ability of local authorities to limit and control the pandemic. Therefore, compared with the direct measurement used in Study 1 (assessing the possibility of being infected by COVID-19 by 5-point scale), we used a variety of questions to ask participants about their perceptions of how effectively local institutions could control and prevent COVID-19. Items included ratings about local medical resource management, the general situation of controlling COVID-19, societal recovery, supply of necessities, the order of societal operation, the surveillance of suspected patients, etc. Each item was reversely coded. The scale was inspired by a scale assessing trust in the healthcare system [32] and a scale measuring the perception of collective community action [33]. Items included, “During the epidemic, the province and city that I live in had sufficient medical supplies”, “During the epidemic, the supply of daily necessities in my province and city was sufficient”, “I think the province and city that I live in is methodically marching towards the end of the outbreak”. Participants were asked to rate the extent to which they agreed with the descriptions from 1 (Strongly disagree) to 7 (Strongly agree). The Cronbach’s α coefficient of a total of 9 items was .90. The higher the mean score, the higher the perceived risk.

### Psychological well-being and social attitudes

#### Emotions

We measured positive and negative emotions from the 20-item version of The Positive and Negative Affect Schedule (PANAS) [34]. We included seven positive emotions (i.e., interested, proud, alert, inspired, determined, attentive, active), and eight negative emotions (i.e., upset, jittery, guilty, scared, hostile, irritable, nervous, afraid). Participants were asked to report the frequency of their different emotions recently using a 7-point scale from 1 (not at all) to 7 (very frequently). The Cronbach’s α coefficients were .83 and .92 respectively.

#### Pressure

Same as Study 1.

#### Self-confidence during the pandemic

We measured participants’ confidence about themselves during the pandemic with a 7-point scale from 1 (not at all) to 7 (very much). The higher score indicated higher confidence in taking effective measures to prevent COVID-19.

#### Organizational trust

We included 5 items respectively to measure participants’ trust in authoritative organizations (authoritative trust) and non-authoritative trust (non-authoritative trust). Authoritative trust included items like trust in central government, central media, local government, researchers engaged in virus research, and healthcare workers; non-authoritative included questions about trust in local media, online social media, neighborhood offices/grassroots organizations, neighborhood committees, charity foundations, and the Red Cross. The mean scores were calculated separately for trust in authority and non-authority. The Cronbach’s α coefficients were .87 and .83 for authority and non-authority respectively.

#### Interpersonal trust

We assessed the participants' trust in others with four items from the Interpersonal Trust Scale [35] (e.g., “Others may take advantage of me if I do not maintain vigilance.”), and three items from the Generalized Trust Scale [36] (e.g., “Most people were honest,” “Most people were trustworthy,”). Participants were asked to rate their attitudes from 1 (Strongly disagree) to 7 (Strongly agree). After reverse coding the two items from Rotter’s (1968), a higher score indicates a higher level of trust toward other people. The Cronbach’s α coefficient was .74.

#### COVID-19 prevention confidence

We asked participants about their confidence in their families, places where they worked, where they lived, local and central government, medical workers, China, and other countries on a 7-point scale from 1 (not at all) to 7 (very much). We calculated the mean score of all items as the indicator of people’s confidence in COVID-19 prevention. A higher score indicates a higher level of confidence. The Cronbach’s α coefficient was .83.

#### Demographic and other variables

We collected participants’ general demographic information, including gender, age, education levels, and socioeconomic status (SES) (using the ladder scale [37]). Additionally, we assessed participants’ perceptions of restriction during the pandemic in the last week and from January to March (corresponding to Study 1 sampling time) to demonstrate the difference in time between Studies 1 and 2. Items included, “During the epidemic period (mainly from January to March), how do you think your daily life is restricted?” “In the last week, how much do you think your daily life is restricted?”. People’s sense of restriction in the last week was significantly lower than that from January to March (covered our sampling time in Study 1) (M_difference_ = − 1.87, SD_difference_ = 1.85, *t* (306) = − 17.66, *p* < .001), but there was no significant difference between sense of restriction in the last week and sense of restriction before the pandemic (M_difference_ = −.01, SD_difference_ = 2.15, *t* (306) = −.11, *p* = .92). Therefore, we considered that Study 2, which took place during the recovery period of the pandemic, took place during a different cultural climate than Study 1 (during the outbreak period).

### Study 3

#### Perceived social norms

We adopted the same measures as Study 2. The scale of perceived social norms during the pandemic was 6-point (1 = Not agree at all, 6 = Totally agree). We transformed the scale into 7 points. The Cronbach’s α coefficients were .54 in the American sample and .45 in the Canadian sample. For the normal-time social norm perception (i.e., TL), we used the same Latitude vs. Constraint in Daily Life Scale as Study 2 [31]. The Cronbach’s α coefficients were .41 in the American sample and .57 in the Canadian sample. The higher the score, the more constraints people perceived in their daily life.

#### Pandemic risk perception

We used the same Risk Perception Scale in this study. All 9 items were translated and then back-translated by two proficient English users. The Cronbach’s α coefficients were .82 and .81 in the American and Canadian samples respectively.

### Psychological well-being and social attitudes

#### Emotions

As in Study 2, we used items from the 20-item version of The Positive and Negative Affect Schedule (PANAS) [34] to measure participants’ positive and negative emotions in the last week. The 5-point scale consisted of four positive emotions (i.e., proud, alert, inspired, active) and six negative emotions (i.e., upset, hostile, irritable, ashamed, nervous, and afraid). We used the mean scores of positive emotions and negative emotions as indicators for emotions. The Cronbach’s α coefficients were .81 and .84 for positive and negative emotion in American and .75 and .82 in Canadian.

#### Self-prevention confidence

Same as Study 2.

#### Organizational trust

Same as Study 2. The authoritative trust Cronbach’s α coefficients were .75 and .68 in the American and Canadian samples respectively; the non-authoritative trust Cronbach’s α coefficients were .88 and .75 respectively.

#### Interpersonal trust

We used the same structure as Study 2 but deleted one item (“You should better be careful when interacting with strangers unless they provide evidence that proves their trustworthiness.”) due to consideration for internal reliability of the two samples. The Cronbach’s α coefficients in the American and Canadian samples were .85 and .82 respectively.

#### COVID-19 prevention confidence

Same as Study 2. The Cronbach’s α coefficients in the American and Canadian samples were .81 and .84 respectively.

#### Demographics and other covariates

Besides age, gender, job, and SES, we also asked participants to report their political affiliation (“In general, what is your political affiliation?”) and the population mobility in their community and province/city by 7-point scale respectively, such as “What is the proportion of the recurrent population (e.g., temporary residents, passengers, and in-transit population) in your community?”,” What is the proportion of the recurrent population (e.g. temporary residents, passengers, and in-transit population) in your community?”

We provided the content validity index of the scales that we made in the Supplementary Materials (Table S2).

### Statistical analysis

First, we tested whether individual perceived risk was associated with people’s psychological well-being and social attitudes by Pearson *r* correlation. Correlation analyses were also used to test for the association between perceived pandemic norm strength and psychological outcomes. Finally, if the above pairwise correlations between pandemic norms and risk, and between risk and psychological variables, were supported, we performed mediation analyses to examine whether perceived pandemic norm strength was linked with psychological outcomes through perceived pandemic risk. The mediation analysis used SPSS PROCESS macro version 3.5, Model 4 [38]. We used the 95% confidence interval (CI) of the indirect effect as the criterion to identify the mediating effect. We set the number of bootstrapped samples as 5000 to ensure stable estimates each time. We additionally ran the power analysis on all mediational models and provided the information in the Supplementary Methods.

## Results

### Study 1

First, we tested whether individual perceived risk was associated with psychological well-being and social attitudes by Pearson *r* correlation. As Table 1 illustrates, higher levels of risk perception correlated with less positive emotions (*r* (1148) = −.14, *p* < .001) like happy and hopeful; more psychological pressure (*r* (1108) = .25, *p* < .001) and more negative emotions (e.g., anxious, *r* (1151) = .34, *p* < .001). For trust in groups and organizations, higher levels of risk correlated with lower trust in authoritative organizations (*r* (1146) = −.19, *p* < .001) but not with trust in non-authoritative organizations (*r* (1152) = −.02, *p* = .483). Taken together, if people perceived greater pandemic risk, they tended to report worse well-being and less trust in authoritative groups and organizations.

**Table 1:** Mean, Standard Deviation (SD) and Correlations in Study 1

Variables	N	Mean	SD	1	2	3	4	5	6	7	8
1.Cultrual TL	1159	4.83	1.45	\							
2.Pandemic Norm	1160	5.50	1.38	.45***	\						
3.Risk	1158	2.61	1.16	−.14***	−.13***	\					
4.Positive Emotions	1150	1.31	.64	.13***	.04	−.14***	\				
5.Negative Emotions	1153	.82	.62	−.12***	−.12***	.34***	−.19**	\			
6.Pressure	1110	47.98	26.86	−.07*	−.11***	.25***	−.23**	.56**	\		
7.Trust Authority	1148	3.60	.70	.29***	.18***	−.19***	.31***	−.30**	−.20**	\	
8.Trust Non-Authority	1154	2.69	.59	.01	.02	−.03	−.01	.04	.02	.12**	\

Cultural TL = Generalized TL; Pandemic Norm = individual perceived social norm during the pandemic; Risk = the possibility of being infected with COVID-19; Trust Authority/Non-Authority = two dimensions of organizational trust (trust in authoritative/non-authoritative organizations respectively)

*p < .05. **p < .01. ***p < .001. †*p* < .10

Next, correlation analyses were used to test the association between perceived pandemic norm strength and psychological outcomes. As Table 1 showed, we found that people who perceived stronger norms during the pandemic felt less pandemic risk (*r* (1156) = −.13, *p* < .001). Stronger pandemic norms were correlated with less negative emotion (*r* (1151) = −.12, *p* < .001) and lower levels of psychological pressure (*r* (1108) = −.11, *p* < .001), but there was no relationship with average positive emotions (*r* (1148) = .04, *p* = .221) or specific positive emotions like happy (*r* (1150) = .01, *p* = .847) or hopeful (*r* (1150) = .05, *p* = .098). For attitudes toward other people and organizations, stronger pandemic norms corresponded with higher levels of trust in authoritative groups and organizations (*r* (1146) = .18, *p* < .001) but not with non-authoritative organizations (*r* (1152) = −.02, *p* = .483). We also found associations between pandemic norm strength and cultural TL (Table S3), even when controlling for covariates (incl., daily cumulative number of COVID-19 cases, provincial population, and environmental risk) (Table S4). As previous research found that there are general psychological differences between people living in tighter vs. looser cultures [39], we performed partial correlations between pandemic norm strength and a set of outcome variable controlling for cultural TL and found most of the results remained (Table S5).

**Fig. 1:**
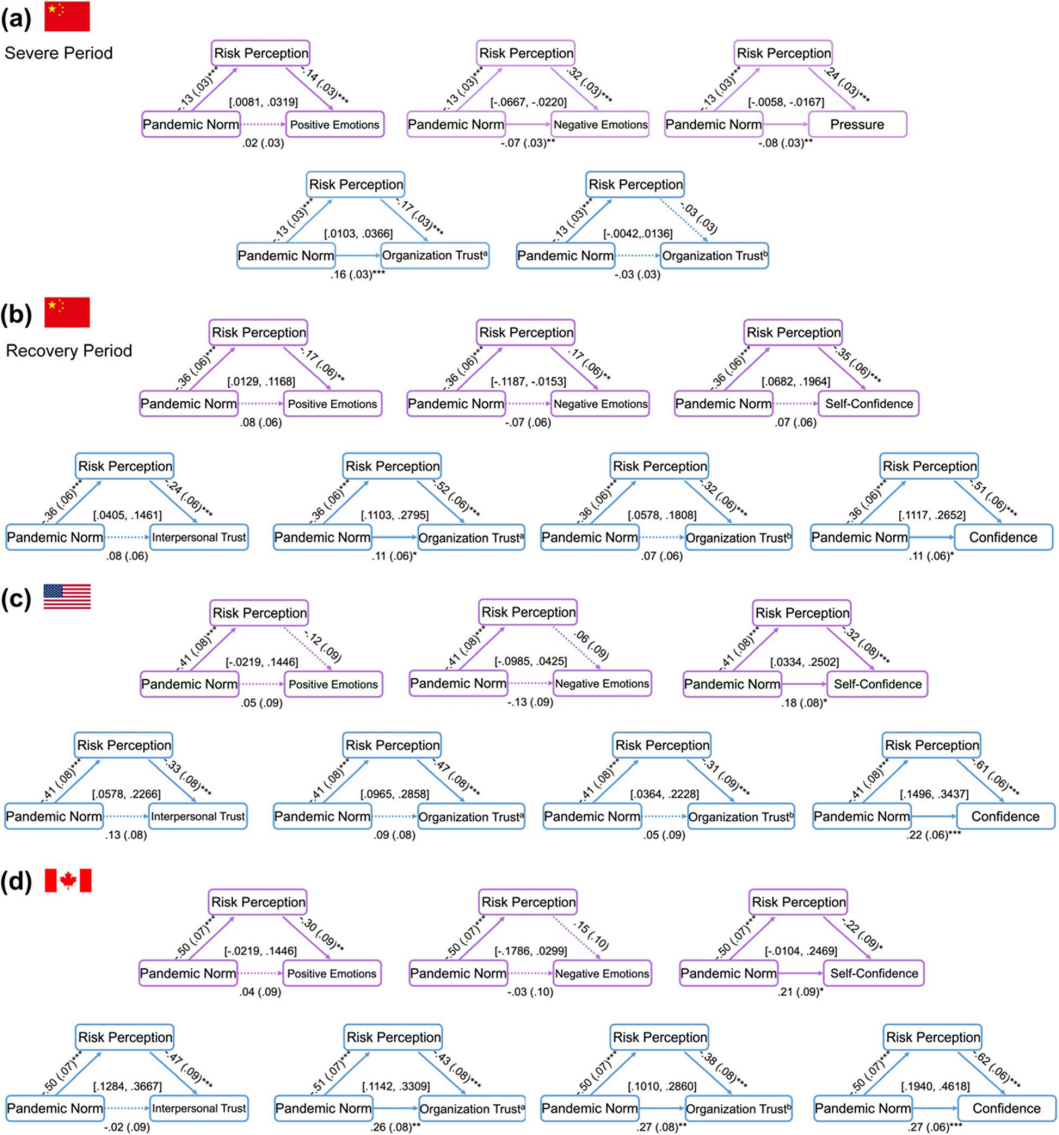
Mediation Models Predicting Psychological Outcomes. *Note.* The figure shows the mediation role of risk perception in the relationship between perceived social norms and psychological well-being and social attitudes for Study 1 (**a**) (Chinese sample at the severe period of the COVID-19), Study 2 (**b**) (Chinese sample at the recovery period) and Study 3 (**c**) (American sample at the severe period) and (**d**) (Canadian sample at the severe period) respectively. All variables were standardized before the mediation analyses. Pandemic Norm = individual perceived social norm during the pandemic; Risk = the possibility of being infected with COVID-19; Positive/Negative Emotions = positive/negative emotions; Pressure = psychological pressure during the pandemic; Self-Confidence = confidence in self during the pandemic; Interpersonal Trust = generalized interpersonal trust; Organization Trust^a^ = trust in authoritative groups and organizations; Organization Trust^b^ = trust in non-authoritative groups and organizations; Confidence = confidence in other people, places, governments and other institutions. Standardized regression coefficients (β) are presented above the arrows. Bold lines represent significant paths. The 95%CI values presented above the bottom line indicate the indirect effect of risk perception exist within the influence of pandemic norms on psychological outcomes. Statistical significance: *p < 0.05; **p < 0.01; ***p < 0.001

To test whether perceived strength of social norms during the pandemic was associated with risk perception and individual-level psychological well-being and social attitudes, we conducted mediation analyses (Fig. 1a). The results showed that, through reduced risk perception, perceived stronger pandemic norm strength was associated with higher positive emotion (B = .02, 95% CI = [.0081, .0319]), lower negative emotion (B = −.04, 95% CI = [−.0667, −.0220]), and less pressure (B = −.03, 95% CI = [−.0522, −.0167]). Through reducing risk perception, perceived pandemic norm strength was also associated with greater trust in authoritative groups or organizations (e.g., government, WHO) (B = .02, 95% CI = [.0103, .0366]) but not in non-authoritative organizations (B = .01, 95% CI = [−.0042, .0136]). Finally, our power analyses for the mediation models reached over .90.

### Study 2

We first replicated key associations involving risk perception. In Study 2, the updated indicator of risk perception was correlated with the single item of perceived susceptibility that was used in Study 1 (*r* (296) = .27, *p* < .001), and the result of the new risk indicator was consistent with the result linked with the previous indicator (Table S6). In particular, people who perceived more risk during the pandemic showed less frequent positive emotions (*r* (296) = −.20, *p* = .001), more frequent negative emotions (*r* (296) = .19, *p* = .001), more pressure (*r* (296) = .10, *p* = .09), and lower levels of self-confidence (*r* (296) = −.37, *p* < .001), interpersonal trust (*r* (296) = −.27, *p* < .001), organizational trust (authoritative: *r* (296) = −.56, *p* < .001); non-authoritative: (*r* (296) = −.34, *p* < .001), and confidence during the pandemic *r* (296) = −.55, *p* < .001).

It was unclear whether stronger pandemic norms during the recovery period still brought about better psychological outcomes. The results of Study 2 validated our findings in Study 1 (Table S6). Perceived strength of pandemic norms was negatively correlated with perceived pandemic risk (*r* (296) = −.35, *p* < .001). For psychological well-being, stronger pandemic norms during the pandemic were associated with more positive emotions (*r* (305) = .14, *p* = .018), more self-confidence (*r* (296) = .19, *p* = .001) and less negative emotions (*r* (305) = −.12, *p* = .036), but not correlated with pressure (*r* (305) = −.01, *p* = .805). For social attitudes, stronger pandemic norms were associated with higher levels of interpersonal trust (*r* (305) = .16, *p* = .005), organizational trust (authoritative: *r* (296) = .29, *p* < .001; non-authoritative: *r* (296) = .18, *p* = .001), and confidence (*r* (305) = .29, *p* < .001). Perceived strength of social norms before the pandemic (e.g., cultural TL) was also associated with positive psychological outcomes (Table S6).

As Fig. 1b illustrated, mediation results of Study 2 showed that, through lower perceived risk, the perceived strength of pandemic norms was associated with greater positive emotions and self-confidence in COVID-19 prevention and lower negative emotions; perception of stronger social norms was also positively associated with interpersonal trust, organizational trust, and confidence in other people, places, governments, and organizations. Our power analyses for the mediation models reached over .80 (Supplementary Methods). This result was also consistent with the result measured with the original single item assessing risk perception (Fig. S1).

### Study 3

In Study 3, data from the United States and Canada were not significantly different across all measures but perceptions of pandemic norm strength (Table S7). We thus analyzed these countries  separately and also reported merged results in the main text. Consistent with Studies 1 and 2, the results of Study 3 showed that people who perceived higher risk during the pandemic showed less frequent positive emotions, more frequent negative emotions, lower self-confidence, and lower levels of interpersonal trust, organizational trust, and confidence in other people and organizations. In general, risk perception was associated with worse well-being and less positive attitudes in American (Table S8) and Canadian (Table S9).

In Study 3, within loose cultures, we also found that stronger perceived pandemic norms were correlated with lower perceived risk both in American and Canadian samples (All: *r* (145) = −.47, *p* < .001; US: *r* (145) = −.41, *p* < .001; Canada: *r* (137) = −.50, *p* < .001). Similarly, the relationship between pandemic norms and psychological well-being and social attitudes replicated the results shown in Chinese samples. Across the two loose culture samples, the perception of stronger pandemic norms was associated with more positive emotions (*r* (285) = .12, *p* = .036), less negative emotions (*r* (286) = −.14, *p* = .018) and higher level of self-confidence (*r* (286) = .32, *p* < .001). As for social attitudes, stronger perceived pandemic norms were correlated with higher levels of interpersonal trust (*r* (286) = .26, *p* = <.001), trust in both authoritative organizations (*r* (285) = .40, *p* < .001) and non-authoritative groups and organizations (*r* (286) = .30, *p* < .001), and confidence in other people and organizations during the pandemic (*r* (286) = .55, *p* < .001). The separate results for American and Canadian samples are provided in Tables S8–S9. When cultural TL was controlled by partial correlation, the above results were all supported (Table S10).

As Fig. 1c and d showed, the results of the loose cultures replicated the findings in the Chinese sample. Through reducing perceived pandemic risk, stronger perceived pandemic-related social norms was associated with higher  positive emotions and self-confidence in COVID-19 prevention, lower negative emotions, higher levels of interpersonal and organizational trusts, and more confidence in other people, places, governments, and other institutions.

## Discussions

COVID-19 changed social norms around the world [40]. However, insufficient attention has been paid to the relationship between the emerging pandemic norms and people’s psychological well-being and attitudes during the COVID-19 pandemic. The current study fills this gap by showing that the strength of pandemic norms, as relative but distinct from TL (which describes the generalized strength of social norms across domains of life), is positively associated with individual psychological well-being and attitudes toward other people and organizations during the pandemic (Study 1, 2, & 3). People who perceive stronger pandemic norms report better psychological well-being, e.g., more frequent positive emotions and less frequent negative emotions (all studies), less psychological pressure (Study 1 & Study 2) and more self-confidence (Study 2 & Study 3); and report more positive social attitudes, e.g., more trust in both authoritative and non-authoritative organizations (all studies), more generalized interpersonal trust (Study 2 & Study 3), and higher levels of confidence in other peoples and organizations (Study 2 & Study 3). Further, pandemic norm strength is negatively related to individual-level risk perception about COVID-19 in both tight (China) (Study 1 & 2) and loose cultures (America and Canada) (Study 3). Additionally, stronger perceived social norms during the pandemic are linked with psychological well-being and attitudes through a lower level of perceived pandemic risk in both culturally tight and loose countries.

Previous literature has suggested that tight cultures are more prepared to deal with ecological and societal threats because tighter social norms improve social coordination at the group level [15, 18]. Now, there is growing evidence that stronger social norms also have a positive psychological effect at the individual level. For example, a research on 1827 Chinese adolescents found that increased risk perception of COVID-19 was associated with more emotional disorders, i.e., anxiety and depression, but that this link was weaker among people who perceived more cultural tightness [41]. They further found that tight culture alleviated the psychological disorders by enhancing perceived protection efficacy. Consistent with their findings, across three studies, we found that increased risk perception and tighter perceived norms were each associated with psychological outcomes and prosocial attitudes. Whereas this previously published study failed to find the link between cultural TL and risk perception among Chinese adolescents, we found medium-to-strong negative associations between pandemic norms and risk perception across the three studies (β = − 0.13 in Study 1; − 0.36 in Study 2; − 0.41 and − 0.50 in Study 3). One possible explanation for this difference in findings is that the pandemic norm measure in the current study is more relevant to the COVID-19 situation and linked with behaviors geared toward protecting individuals from infection [42].

Tighter norms are theorized to have evolutionary benefits in promoting society security and stability in ancient times [17]. Consistently, our study and previous research [23] both show that stronger social norms were linked to positive outcomes at the group and individual level during the COVID-19 pandemic. The current study reveals the paramount importance of studying the strength of social norms, and how this strength changes over time. For example, routinely practicing emergency drills for pandemic prevention and related security drills can help control the spread of pandemics and prevent social turmoil.

Our research also distinguished between the perceived risk of COVID-19 and the perceived strength of social norms during COVID-19. Some previous studies have found that the perceived risk of COVID-19 is associated with more negative emotions and higher degrees of depression, and declined trust in public information [11, 43]; however, another study found that people in the pandemic reported higher trust in science, ﻿politicians, and police, and higher levels of patriotism, but higher rates of mental distress compared to people in the pre-lockdown pre-pandemic group [44]. Potentially resolving this discrepancy, this study provides a new insight that stronger pandemic norms correspond with lower risk perception, which ameliorates negative feelings and attitudes in both tight and loose cultures. Previous research also found that the impact of COVID-19 has a negative association with psychological well-being but a positive association with social attitudes [44]. Thus, we do not only measure people’s psychological well-being (i.e., emotions, pressure, and self-related confidence), but also attitudes toward other people, groups and organizations. Trust in organizations reflects the interaction between individuals, groups, and government agencies during the pandemic. Government agencies may benefit from paying attention to how people perceive tightened social norms, and COVID-19 risk in order to maintain a better relationship with the people, and facilitate social harmony and stability.

### Limitations

Despite the intriguing findings, the current work has some limitations. First, we focus on the strength of pandemic norms, an extension of TL and its psychological influence, instead of measuring TL directly. We do not intend to exclude the possible role of TL. In fact, our results show that pandemic norm strength seems to show a positive correlation with TL (See Supplementary Results). However, more evidence is needed to investigate the influence of TL on pandemic norm strength. Future research could extend our results by investigating the association between emerging norms and long-term norms. Second, the current study is a cross-sectional study which cannot assess causality. Caution is needed when interpreting our results. Third, given that the data of non-responders were not available, we cannot reliably estimate the non-response bias. Future work is suggested to compare responders with non-respondents on the measures of interest. Finally, as the pandemic situation changes, we assume that the strength of pandemic norms also changes across time, but we have not investigated the psychological influence of changes in pandemic norm strength. Future research can use multiple methods, such as qualitative analysis, to analyze the changes of the pandemic norms in countries and regions, e.g., the trends of social norms over time, the inflection points of the COVID-19 pandemic, and their associations with the public’s psychological characteristics. These, we believe, will help extend current findings and enlighten future pandemic management.

## Conclusion

The current study deepens our understanding of COVID-19’s influence on both psychological well-being and social attitudes. The current research provides a cultural psychology perspective to examine the changes in social norms brought about by COVID-19, the relationship between social norm strength and perceived risk, and the association between the two and both psychological well-being (emotions, psychological pressure, and self-confidence) and social attitudes (interpersonal trust, organizational trust, and confidence in others and organizations). This complements previous literature by illustrating the potential benefits of tight norms in managing public health crises. For example, tightening social norms could also improve psychological well-being during the pandemic. Policy makers may need to specifically target people are less sensitive to social norms and their dynamic changes.

## Supplementary Information

**Additional file 1.**

## Data Availability

De-identified data and code of the present study is available upon request to the corresponding author Prof. Mu.
