# Supplementary material for: Perception of strong social norms during the COVID-19 pandemic is linked to positive psychological outcomes
Source: BMC Public Health. 2022 Jul 22;22:1403. doi: 10.1186/s12889-022-13744-2 (PMC9305059; doi:10.1186/s12889-022-13744-2)
Supplement: Supplementary file 1 — Additional file 1. [file 12889_2022_13744_MOESM1_ESM.docx]

**ONLINE SUPPLEMENT MATERIAL**

**Perception of Strong Social Norms during the COVID-19 Pandemic is Linked to Positive Psychological Outcomes**

Shuang Liu^1^, Jiajia Zhu^1,2^, Yutong Liu^1,2^, Danica Wilbanks^3^, Joshua Conrad Jackson^4^, Yan Mu^1,2,*^

1 CAS Key Laboratory of Behavioral Science, Institute of Psychology, Chinese Academy of Sciences, Beijing, 100101, China

2 Department of Psychology, University of Chinese Academy of Sciences, Beijing, 100049, China

3 Department of Psychology and Neuroscience, University of North Carolina at Chapel Hill, NC 27599, US

4 Kellogg School of Management, Northwestern University, IL 60208, US

**Running title**: Social norms and COVID-19

***Correspondence:** **Prof. Yan Mu**

**Email:** [muy@psych.ac.cn](mailto:muy@psych.ac.cn)

**Address:** 16 Lincui Road, Chaoyang District, Beijing 100101, China

**Brief Introduction of Supplementary Material**

We supplement information and extended results that are not presented in the main text, including:

Supplementary Methods

Supplementary Results

Supplementary Tables

Supplementary Figure

**Supplementary Methods**

**Sample Size and Power Analysis**

**Study 1**

We used the widely accepted method — Monte Carlo Power Analysis for Indirect Effects (Schoemann et al., 2017) to determine the sample size. For a relatively large-scale survey, and our required power level at 0.80, we set a minimum sample size of 100, a maximum sample size of 1000, and a step size of 100. We hypothesized the direction of pairwise correlations in our main text — Introduction part and pre-set all correlation coefficients at a moderate level (±.5). Thus, we obtained our ideal sample is at least 100. We distributed our online questionnaire after the automatic filter was set, however, we got a sample size exceeding the required one. Due to all participants volunteering to participate without compensation, we kept all valid data.

We also conducted post hoc power analyses for the mediation models. The same method by Schoemann et al. (2017) was used according to our factual valid sample (*N* = 1161) and correlation matrixes. We set the number of replications as 5000, Monte Carlo Draws per Rep as 20000 according to the published recommendations, and the confidence interval width as 95%. Results showed that the power of our five mediation models all exceeded .99, except .20 for the unsupported model with trust in authoritative organizations as outcome variables.

**Study 2**

According to our previous calculation for sample size in Study 1, our ideal sample size for testing indirect effects is at least 100.

We conducted post hoc power analyses for the mediation models. We input the actual sample as 307, and set the number of replications as 5000, Monte Carlo Draws per Rep as 20000 according to the published recommendations, and the confidence interval width as 95%. Results showed that the power of our eight mediation models all exceeded .80, except .44 for one unsupported model predicting pressure.

**Study 3**

According to our previous calculation for sample size in Study 1, our ideal sample size for testing indirect effects is at least 100.

We also conducted post hoc power analyses for our mediation models. We input the actual sample as 149 and 139 respectively for Americans and Canadians, and set the number of replications as 5000, Monte Carlo Draws per Rep as 20000 according to the published recommendations, and the confidence interval width as 95%. Results showed that the power of our seven mediation models all exceeded .90 in the American sample and all exceeded .62 in the Canadian sample.

**Supplementary Results**

**The relevance between tightness before and after the COVID-19 outbreak (cultural TL and pandemic norms).** In our three sub-studies, TL was positively correlated with pandemic norms. Namely, the tighter the perceived social norms in general, the stronger the perceived pandemic-related norms. This result was supported across tight and loose cultures. We then performed a hierarchical linear regression to control the effects of provincial population (both population number and population density), objective COVID-19 risk (daily cumulative number of COVID-19 cases), and subjective risk (COVID-19 severity perception, subjective COVID-19 distance, and risk in the community and city/town). After considering these covariates, TL still predicted the strength of pandemic norms. See Table S1-S2, Table S5-S6, and Table S9-S12.

**Supplementary Tables**

**Table** **S1** Variable differences in demographics, cultural TL, pandemic norms, and mentalities between two-round data (Study 2)

| Measure | | 1^st^ Round (N = 205) | | | | 2^nd^ Round (N = 102)) | | | | *t*_(1,305)_ | |  |
| --- | --- | --- | --- | --- | --- | --- | --- | --- | --- | --- | --- | --- |
|  |  | M | | SD | | M | | SD | |  |  |  |
| Demographics | Gender | | 1.62 | | .49 | | 1.62 | | .49 | | .03 | |
|  | Age | | 24.46 | | 7.09 | | 25.05 | | 7.24 | | -.68 | |
|  | Education | | 5.17 | | .88 | | 4.94 | | .79 | | *2.30* | |
|  | SES | | 5.05 | | 1.53 | | 5.08 | | 1.43 | | -.16 | |
| Social Norms | Daily TL | | 4.86 | | .72 | | 4.99 | | .80 | | -1.38 | |
|  | Generalized TL | | 4.61 | | .80 | | 4.63 | | .75 | | -.20 | |
|  | Pandemic Norms | | 5.30 | | .90 | | 5.25 | | .85 | | .40 | |
|  | Norm Differences | | .44 | | .85 | | .27 | | .89 | | 1.59 | |
| Risk | Risk (one-item) | | 1.85 | | 1.05 | | 1.83 | | 1.12 | | .18 | |
|  | Risk (scale) | | 2.17 | | .87 | | 1.97 | | .76 | | 1.92† | |
| Mentality | PA | | 3.87 | | 1.09 | | 4.03 | | 1.09 | | -1.21 | |
|  | NA | | 2.90 | | 1.27 | | 2.68 | | 1.37 | | 1.42 | |
|  | Pressure | | 5.74 | | 2.28 | | 5.25 | | 2.46 | | 1.72† | |
|  | Interpersonal Trust | | 4.65 | | .79 | | 4.74 | | .80 | | -.95 | |
|  | Trust Authority | | 6.06 | | .93 | | 6.02 | | .94 | | .37 | |
|  | Trust Non-Authority | | 4.59 | | 1.17 | | 4.73 | | 1.24 | | .97 | |
|  | Self-Confidence | | 6.21 | | .98 | | 6.12 | | 1.06 | | .75 | |
|  | Confidence (others) | | 5.84 | | .77 | | 5.83 | | .77 | | .14 | |

*Note*. SES = socioeconomic status; Daily TL/Generalized TL = two measurements of cultural TL; Risk (scale) = the mean score of risk perception scale; Risk (one-item) = the possibility of being infected with COVID-19; Confidence (self) = self-confidence during the pandemic; Confidence (others) = mean score of confidence in people (families, medical workers), working place/school, the central/local government and countries (China and other countries); Trust Authority/Non-Authority = two dimensions of organizational trust (trust in authoritative/non-authoritative organizations respectively); Norm Difference = the social norm difference of tightness between pandemic and TL by pandemic norms minus daily TL. † *p*<.10

**Table S2. The Content Validity Index of the scales used in the current studies**

| Items of the COVID-19 Prevention Confidence scale | Expert 1 | Expert 2 | Expert 3 | Expert 4 | Expert 5 | Expert 6 |  | Experts in agreement | I-CVI | UA |
| --- | --- | --- | --- | --- | --- | --- | --- | --- | --- | --- |
| 1 Your families | 1 | 1 | 1 | 1 | 1 | 1 |  | 6 | 1 | 1 |
| 2 Places where you work | 1 | 1 | 1 | 1 | 1 | 1 |  | 6 | 1 | 1 |
| 3 Places where you live | 1 | 1 | 1 | 1 | 1 | 1 |  | 6 | 1 | 1 |
| 4 Localgovernment | 1 | 1 | 1 | 1 | 1 | 1 |  | 6 | 1 | 1 |
| 5 Central government | 1 | 1 | 1 | 1 | 1 | 1 |  | 6 | 1 | 1 |
| 6 Healthcare workers | 1 | 1 | 1 | 1 | 1 | 1 |  | 6 | 1 | 1 |
| 7 China | 1 | 1 | 1 | 1 | 1 | 1 |  | 6 | 1 | 1 |
| 8 Other countries | 1 | 1 | 1 | 1 | 1 | 1 |  | 6 | 1 | 1 |
|  |  |  |  |  |  |  |  | **S-CVI/Ave** | 1 |  |
| **Proportion Relevance** | 1 | 1 | 1 | 1 | 1 | 1 |  | **S-CVI/UA** |  | 1 |
| **Average proportion of items judged as relevance across the 6 experts** | | | | |  |  | 1 |  |  |  |
|  |  |  |  |  |  |  |  |  |  |  |
|  |  |  |  |  |  |  |  |  |  |  |
| Items of the Pandemic Risk Perception scale (X is the place where you are living in) | Expert 1 | Expert 2 | Expert 3 | Expert 4 | Expert 5 | Expert 6 |  | Experts in agreement | I-CVI | UA |
| 1 During the epidemic, the medical supplies in X are sufficient. | 1 | 1 | 1 | 1 | 1 | 1 |  | 6 | 1 | 1 |
| 2 I can trust the ability of medical workers in X. | 1 | 1 | 1 | 1 | 1 | 1 |  | 6 | 1 | 1 |
| 3 I feel that the epidemic in X has already been controlled effectively. | 1 | 1 | 1 | 0 | 1 | 1 |  | 5 | 0.83 | 0 |
| 4 I think the ways that people in X prevent and control the epidemic are effective. | 1 | 1 | 1 | 1 | 1 | 1 |  | 6 | 1 | 1 |
| 5 I think X is methodically marching towards the end of the outbreak. | 1 | 0 | 0 | 1 | 0 | 1 |  | 3 | 0.5 | 0 |
| 6 I feel that at present X is running as it was before the outbreak. | 1 | 1 | 1 | 0 | 1 | 1 |  | 5 | 0.83 | 0 |
| 7 X can always successfully get through the universal difficulties (e.g. epidemics like COVID-2019). | 1 | 1 | 1 | 1 | 1 | 1 |  | 6 | 1 | 1 |
| 8 During the epidemic, the supplies of life necessities in X are sufficient. | 1 | 1 | 1 | 1 | 1 | 1 |  | 6 | 1 | 1 |
| 9 (Reverse) During the epidemic, the possibility of unemployment is higher than usual. | 1 | 1 | 1 | 1 | 1 | 1 |  |  |  |  |
|  |  |  |  |  |  |  |  | **S-CVI/Ave** | 0.90 |  |
| **Proportion Relevance** | 1 | 0.89 | 0.89 | 0.78 | 0.89 | 1 |  | **S-CVI/UA** |  | 0.56 |
| **Average proportion of items judged as relevance across the 6 experts** | | | | |  |  | 0.91 |  |  |  |
|  |  |  |  |  |  |  |  |  |  |  |

*Note*. The calculation of Content Validity Index referred to [Yusoff, M. S. B. (2019). ABC of content validation and content validity index calculation. *Educational Resource, 11*(2), 49-54.]. All experts rated all items on each questionnaire on a scale of one to four points. The score of 1 indicated that the item was not relevant to the measured domain, the score of 2 indicated that there was a weak correlation, the score of 3 indicated that the item was quite relevant to the measured domain, and the score of 4 indicated that the item was highly relevant to the measured domain. Finally, scores 1 and 2 are re-coded as 0, 3 and 4 as 1. I-CVI = the expert in agreement divided by the number of experts; Universal agreement (UA) = score “1” is assigned to the item that achieved 100% experts in agreement; S-CVI/Ave (based on I-CVI) = the average of I-CVI scores across all items; S-CVI/UA = the average of UA scores across all items.

**Table S3.** Mean, standard deviation (SD) and correlations in Study 1

| Variables | Mean | SD | 1 | 2 | 3 | 4 | 5 | 6 | 7 | 8 | 9 |
| --- | --- | --- | --- | --- | --- | --- | --- | --- | --- | --- | --- |
| 1.Generalized TL | 4.83 | 1.45 | \ |  |  |  |  |  |  |  |  |
| 2.Pandemic Norm | 5.50 | 1.38 | .45*** | \ |  |  |  |  |  |  |  |
| 3.Self Risk | 2.61 | 1.16 | -.14*** | -.13*** | \ |  |  |  |  |  |  |
| 4.Positive Emotions | 1.31 | .64 | .13*** | .04 | -.14*** | \ |  |  |  |  |  |
| 5.Negative Emotions | .82 | .62 | -.12*** | -.12*** | .34*** | -.19** | \ |  |  |  |  |
| 6.Pressure | 47.98 | 26.86 | -.07* | -.11*** | .25*** | -.23** | .56** | \ |  |  |  |
| 7.Trust Authority | 3.60 | .70 | .29*** | .18*** | -.19*** | .31*** | -.30** | -.20** | \ |  |  |
| 8.Trust Non-Authority | 2.69 | .59 | .01 | .02 | -.03 | -.01 | .04 | .02 | .12** | \ |  |
| 9.Norm Difference | .67 | 1.49 | -.56*** | .49*** | .01 | -.10* | .01 | -.04 | -.12** | -.02 | \ |

*Note*. *N* = 1161. Pandemic Norm = individual perceived social norm during the pandemic; Risk = the possibility of being infected with COVID-19; Trust Authority/Non-Authority = two dimensions of organizational trust (trust in authoritative/non-authoritative organizations respectively); Norm Difference = the social norm difference of tightness between pandemic and TL by pandemic norm strengths minus TL. **p*<.05. ***p*<.01. ****p*<.001. †*p*<.10

**Table S4** Hierarchical Regression Results for Predicting Pandemic Norms by TL (Study 1)

|  | Model 1 | | | | Model 2 | | | |
| --- | --- | --- | --- | --- | --- | --- | --- | --- |
| Predictor | *B* | *SE* | *t* | *p* | *B* | *SE* | *t* | *p* |
| (Intercept) | .03 | .03 | 1.11 | .27 | .02 | .03 | .84 | .40 |
| Confirmed cases | -.05 | .03 | -1.53 | .13 | -.03 | .03 | -.98 | .33 |
| Mobility (inflow) | .02 | .08 | .26 | .80 | -.01 | .07 | -.13 | .90 |
| Mobility (outflow) | -.07 | .10 | -.71 | .48 | -.04 | .09 | -.51 | .61 |
| Population | .02 | .07 | .24 | .81 | .01 | .07 | .17 | .87 |
| Population density | -.02 | .04 | -.47 | .64 | -.09 | .04 | -2.45 | .02 |
| Subjective COVID-19 distance | .07 | .03 | 1.93 | .05 | .05 | .03 | 1.76 | .08 |
| Surrounding risk perception | -.06 | .05 | -1.31 | .19 | -.02 | .04 | -.48 | .63 |
| Self-risk perception | -.11 | .05 | -2.30 | .02 | -.07 | .04 | -1.66 | .10 |
| Cultural TL |  |  |  |  | .43 | .03 | 14.47 | <.001 |
| *R*^2^ | .03*** | | | | .20*** | | | |
| *F* | 3.41 | | | | 26.90 | | | |
| *R*^2^-change | \ | | | | .17*** | | | |

*Note*. *N* = 1161. After standardizing continual variables, we entered all variables hierarchically. We placed all covariates including cumulative COVID-19 cases, province-level mobility rate (inflow and outflow), province-level population, population density, psychological distance with COVID-19 and subjective COVID-19 risk perception in Model 1, and entered generalized cultural TL in the Model 2 to exclude the effects of variables in the first model on the dependent variable (i.e., Pandemic norms) and see the true statistical power of independent variable (i.e., TL) in predicting Pandemic norm strength. Variables’ coefficients, standard errors and *t* and *p* value in each model were presented in the table. The model fit measures (*R*^2^, *F* and model significance) and comparisons (*R*^2^-change and its significance) was presented at the bottom of the table.

**Table S5**. Partial Correlations by Controlling TL in Study 1

| Variables | 1 | 2 | 3 | 4 | 5 | 6 | 7 |
| --- | --- | --- | --- | --- | --- | --- | --- |
| 1.Pandemic Norms | \ |  |  |  |  |  |  |
| 2.Risk | -.07* | \ |  |  |  |  |  |
| 3.Positive Emotions | -.02 | -.13*** | \ |  |  |  |  |
| 4.Negative Emotions | -.07* | .33*** | -.17*** | \ |  |  |  |
| 5.Pressure | -.09** | .25*** | -.22*** | .56*** | \ |  |  |
| 6.Trust Authority | .06* | -.17*** | .29*** | -.27*** | -.19*** | \ |  |
| 7.Trust Non-Authority | -.02 | -.03 | -.01 | .04 | .02 | .13*** | \ |

*Note*. Pandemic Norms = individual perceived social norm during the pandemic; Risk = the possibility of being infected with COVID-19; Trust Authority/Non-Authority = two dimensions of organizational trust (trust in authoritative/non-authoritative organizations respectively); Norm Difference = the social norm difference of tightness between pandemic and TL by pandemic norm strengths minus TL.

**p*<.05. ***p*<.01. ****p*<.001. †*p*<.10

**Table** **S6.** Mean, standard deviation (SD), and correlations in Study 2

| Variables | 1 | 2 | 3 | (3) | 4 | 5 | 6 | 7 | 8 | 9 | 10 | 11 | 12 |
| --- | --- | --- | --- | --- | --- | --- | --- | --- | --- | --- | --- | --- | --- |
| 1.Daily TL | \ |  |  |  |  |  |  |  |  |  |  |  |  |
| 2.Pandemic Norms | .45*** | \ |  |  |  |  |  |  |  |  |  |  |  |
| 3.Risk | -.34*** | -.35*** | \ |  |  |  |  |  |  |  |  |  |  |
| (3. Risk (one-item)) | -.11† | -.11† | .27*** | \ |  |  |  |  |  |  |  |  |  |
| 4.Positive Emotions | .37*** | .14* | -.20** | -.04 | \ |  |  |  |  |  |  |  |  |
| 5.Negative Emotions | -.18*** | -.12* | .19** | .34*** | -.21*** | \ |  |  |  |  |  |  |  |
| 6.Pressure | -.12* | -.01 | .10† | .21*** | -.22*** | .51** | \ |  |  |  |  |  |  |
| 7.Interpersonal Trust | .26*** | .16** | -.27*** | -.19** | .23*** | -.28*** | -.28*** | \ |  |  |  |  |  |
| 8.Trust Authority | .35*** | .29** | -.56*** | -.20** | .21** | -.13* | -.11† | .37*** | \ |  |  |  |  |
| 9.Trust Non-Authority | .32*** | .18** | -.34*** | -.08 | .24** | -.13* | -.14* | .33*** | .63** | \ |  |  |  |
| 10.Confidence (self) | .21*** | .19** | -.37*** | -.29*** | .17*** | -.12* | -.13* | .32*** | .52*** | .30*** | \ |  |  |
| 11.Confidence (others) | .34*** | .29*** | -.55*** | -.25*** | .21*** | -.12* | -.12* | .37*** | .75*** | .57*** | .66*** | \ |  |
| 12.Generalized TL | .52*** | .52*** | -.30*** | -.08 | .30*** | -.10† | -.11† | .22*** | .30*** | .25*** | .18** | .29*** | \ |

*Note*. Daily TL/Generalized TL = two measurements of cultural TL; Pandemic Norms = individual perceived social norm during the pandemic; Risk = the mean score of risk perception scale; Risk (one-item) = the possibility of being infected with COVID-19; Trust Authority/Non-Authority = two dimensions of organizational trust (trust in authoritative/non-authoritative organizations respectively); Confidence (self) = self-confidence during the pandemic; Confidence (others) = confidence in other people, places, governments, and organizations.

**p*<.05. ***p*<.01. ****p*<.001. †*p*<.10

**Table** **S7.** Descriptive and Independent-Samples T-Test in TL, pandemic norms, and mentalities in American and Canadian (Study 3)

| Measure | America | | Canada | | *t*_(286)_ |
| --- | --- | --- | --- | --- | --- |
|  | *M* | *SD* | *M* | *SD* |  |
| TL | 3.74 | .73 | 3.85 | .69 | -1.23 |
| Pandemic Norms | 4.71 | 1.03 | 5.14 | .87 | -3.80*** |
| Risk | 5.55 | 2.40 | 5.52 | 2.10 | .12 |
| Positive Emotions | 2.72 | .98 | 2.63 | .87 | .80 |
| Negative Emotions | 1.93 | .83 | 1.90 | .73 | .23 |
| Confidence (self) | 5.70 | 1.27 | 5.81 | 1.20 | -.74 |
| Trust Authority | 3.27 | .63 | 3.59 | .75 | -3.94*** |
| Trust Non-Authority | 2.80 | .66 | 2.85 | .69 | -.56 |
| Interpersonal Trust | 3.75 | 1.09 | 4.03 | .98 | -2.27 |
| Confidence (others) | 4.31 | .95 | 4.92 | .93 | -5.54*** |
| Norm Difference | .96 | 1.00 | 1.28 | .90 | -2.86 |

*Note*. TL = daily TL; Pandemic Norms = individual perceived social norm during the pandemic; Risk = the mean score of risk perception scale; Trust Authority/Non-Authority = two dimensions of organizational trust (trust in authoritative/non-authoritative organizations respectively); Confidence (self) = self-confidence during the pandemic; Confidence (others) = confidence in other people, places, governments, and organizations; Norm Difference = the difference between pandemic norm and daily TL.

**p*<.05. ***p*<.01. ****p*<.001.

**Table S8.** Correlation results in Study 3 (US)

| Variables | 1 | 2 | 3 | 4 | 5 | 6 | 7 | 8 | 9 | 10 | 11 |
| --- | --- | --- | --- | --- | --- | --- | --- | --- | --- | --- | --- |
| 1. TL | \ |  |  |  |  |  |  |  |  |  |  |
| 2.Pandemic Norms | .39*** | \ |  |  |  |  |  |  |  |  |  |
| 3.Risk | .05 | -.41*** | \ |  |  |  |  |  |  |  |  |
| 4.Positive Emotions | .19* | .11 | -.15† | \ |  |  |  |  |  |  |  |
| 5.Negative Emotions | -.10 | -.16* | .12 | -.34*** | \ |  |  |  |  |  |  |
| 6.Confidence (self) | -.04 | .31*** | -.40*** | .14 | -.06 | \ |  |  |  |  |  |
| 7.Trust Authority | .39*** | .29*** | -.51*** | .25** | -.09 | .29*** | \ |  |  |  |  |
| 8.Trust Non-Authority | .23*** | .18* | -.33*** | .21* | -.02 | .36*** | .62*** | \ |  |  |  |
| 9.Interpersonal Trust | .32*** | .26** | -.39*** | .21* | -.20* | .15† | .53*** | .46*** | \ |  |  |
| 10.Confidence (others) | .34*** | .47*** | -.71*** | .29*** | -.28** | .49*** | .65*** | .46*** | .48*** | \ |  |
| 11.Norm Difference | -.33*** | .74*** | -.23** | -.02 | -.10 | .36*** | .02 | .02 | .03 | .25** | \ |

*Note*. TL = daily TL; Pandemic Norms = individual perceived social norm during the pandemic; Risk = the mean score of risk perception scale; Trust Authority/Non-Authority = two dimensions of organizational trust (trust in authoritative/non-authoritative organizations respectively); Confidence (self) = self-confidence during the pandemic; Confidence (others) = confidence in other people, places, governments and organizations; Norm Difference = the difference between pandemic norm and daily TL.

†*p*<.10. **p*<.05. ***p*<.01. ****p*<.001.

**Table S9.** Correlation results in Study 3 (Canada)

| Variables | 1 | 2 | 3 | 4 | 5 | 6 | 7 | 8 | 9 | 10 | 11 |
| --- | --- | --- | --- | --- | --- | --- | --- | --- | --- | --- | --- |
| 1.TL | \ |  |  |  |  |  |  |  |  |  |  |
| 2.Pandemic Norms | .35*** | \ |  |  |  |  |  |  |  |  |  |
| 3.Risk | .09 | -.50*** | \ |  |  |  |  |  |  |  |  |
| 4.Positive Emotions | .29*** | .19* | -.32*** | \ |  |  |  |  |  |  |  |
| 5.Negative Emotions | -.12 | -.11 | .17† | -.24** | \ |  |  |  |  |  |  |
| 6.Confidence (self) | .22* | .32*** | -.32*** | .12 | -.23** | \ |  |  |  |  |  |
| 7.Trust Authority | .18* | .47*** | -.56*** | .15 | -.26** | .34*** | \ |  |  |  |  |
| 8.Trust Non-Authority | .32*** | .46*** | -.52*** | .23** | -.20* | .27** | .70*** | \ |  |  |  |
| 9.Interpersonal Trust | .01 | .21* | -.46*** | .25** | -.30*** | .13 | .47*** | .42*** | \ |  |  |
| 10.Confidence (others) | .19* | .58*** | -.76*** | -.05 | -.20* | .56*** | .64*** | .53*** | .39*** | \ |  |
| 11.Norm Difference | -.43*** | .70*** | -.33*** | -.05 | -.01 | .14 | .32*** | .21* | .22* | .41*** | \ |

*Note*. TL = daily TL; Pandemic Norms = individual perceived social norm during the pandemic; Risk = the mean score of risk perception scale; Trust Authority/Non-Authority = two dimensions of organizational trust (trust in authoritative/non-authoritative organizations respectively); Confidence (self) = self-confidence during the pandemic; Confidence (others) = confidence in other people, places, governments and organizations; Norm Difference = the difference between pandemic norm and daily TL.

†*p*<.10. **p*<.05. ***p*<.01. ****p*<.001.

**Table S10.** Partial Correlations by Controlling TL in Study 3

| Variables | 1 | 2 | 3 | 4 | 5 | 6 | 7 | 8 | 9 |
| --- | --- | --- | --- | --- | --- | --- | --- | --- | --- |
| 1.Pandemic Norms | \ | -.47*** | .09 | -.08 | .25** | .44*** | .40*** | .24** | .54*** |
| 2.Risk | -.36*** | \ | -.27** | .15† | -.28** | -.54*** | -.48*** | -.47*** | -.75*** |
| 3.Positive Emotions | .04 | -.09 | \ | -.20* | .06 | .09 | .14 | .21* | .19* |
| 4.Negative Emotions | -.14† | .09 | -.31*** | \ | -.22** | -.24** | -.18* | -.31*** | -.19* |
| 5.Confidence(self) | .38*** | -.42*** | .13 | -.05 | \ | .31*** | .22* | .14 | .52*** |
| 6.Trust Authority | .18* | -.44*** | .18* | -.04 | .31*** | \ | .70*** | .49*** | .63*** |
| 7.Trust Non-Authority | .11 | -.27*** | .16† | .01 | .37*** | .59*** | \ | .44*** | .51*** |
| 8.Interpersonal Trust | .16* | -.33** | .15† | -.18* | .16† | .47*** | .42*** | \ | .42*** |
| 9.Confidence(others) | .41*** | -.67*** | .21* | -.24** | .52*** | .59*** | .41*** | .41*** | \ |

*Note*. The results for the American sample (n = 149) are shown above the diagonal. The results for the Canadian sample (n = 139) are shown below the diagonal. TL = daily TL; Pandemic Norms = individual perceived social norm during the pandemic; Risk = the mean score of risk perception scale; Trust Authority/Non-Authority = two dimensions of organizational trust (trust in authoritative/non-authoritative organizations respectively); Confidence (self) = self-confidence during the pandemic; Confidence (others) = confidence in other people, places, governments, and organizations; Norm Difference = the difference between pandemic norm and daily TL.

**p*<.05. ***p*<.01. ****p*<.001.

Supplementary Figure.

**Figure S1**. Mediation Results in Study 2**
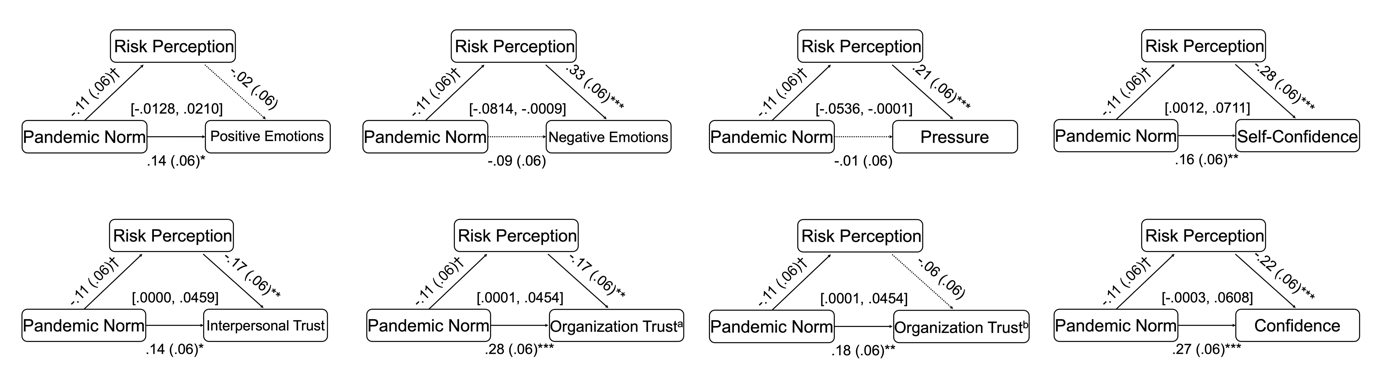
**

*Note.* The figure shows the mediation role of risk perception in the relationship between pandemic norm strength and a set of mentality-related variables in Study 2. Pandemic Norm = social norms perceived during the COVID-19 pandemic; Pressure = psychological pressure during the pandemic; Organization Trust^a^ = trust in authoritative groups and organizations; Organization Trust^b^ = trust in non-authoritative groups and organizations. Standardized regression coefficients (β) examining the strength of Pandemic norms as a mediator of the relationship between mentality-related variables are presented above the arrows. Bold lines represent significant paths. The 95%CI values presented at the top indicate the indirect effect of risk perception. Statistical significance: **p* < 0.05; ***p* < 0.01; ****p* < 0.001.
